# A Cause-of-Death Decomposition of Young Adult Excess Mortality

**DOI:** 10.1007/s13524-018-0680-9

**Published:** 2018-06-04

**Authors:** Adrien Remund, Carlo G. Camarda, Tim Riffe

**Affiliations:** 1 Institute of Demography and Socioeconomics, University of Geneva, Geneva, Switzerland; 2 Institut National d’Études Démographiques, Paris, France; 3 Swiss National Centre of Competence in Research LIVES—Overcoming Vulnerability: Life Course Perspectives, Lausanne, Switzerland; 4 Max Planck Institute for Demographic Research, Rostock, Germany

**Keywords:** Causes of death, Decomposition, Excess mortality, Smoothing, Young adult mortality hump

## Abstract

We propose a method to decompose the young adult mortality hump by cause of death. This method is based on a flexible shape decomposition of mortality rates that separates cause-of-death contributions to the hump from senescent mortality. We apply the method to U.S. males and females from 1959 to 2015. Results show divergence between time trends of hump and observed deaths, both for all-cause and cause-specific mortality. The study of the hump shape reveals age, period, and cohort effects, suggesting that it is formed by a complex combination of different forces of biological and socioeconomic nature. Male and female humps share some traits in all-cause shape and trend, but they also differ by their overall magnitude and cause-specific contributions. Notably, among males, the contributions of traffic and other accidents were progressively replaced by those of suicides, homicides, and poisonings; among females, traffic accidents remained the major contributor to the hump.

**Electronic supplementary material:**

The online version of this article (10.1007/s13524-018-0680-9) contains supplementary material, which is available to authorized users.

## Introduction

Human mortality patterns usually include a brief period of excess mortality in young adult ages, often called the *young adult mortality hump*. Although the hump was first described long ago (Thiele 1871), and most demographers could spontaneously draw its pattern on a napkin, recognizability has not led to extensive theoretical or analytic attention. Consequently, empirical research on the hump has been scarce. One exception is a study on its peak location (Goldstein 2011). Aside from this, research on young adult mortality has not considered the hump pattern as a separate phenomenon from the broader mortality context. Parametric models that do separate the hump have done so for the sake of a better fit to all-cause mortality, but these have not been used to study the hump specifically. We address these shortcomings by first proposing a definition of the mortality hump, operationalized as young adult excess mortality. We then describe a flexible method of measuring the hump by age and causes of death based on a nonparametric shape decomposition of mortality.

**Fig. 1:**
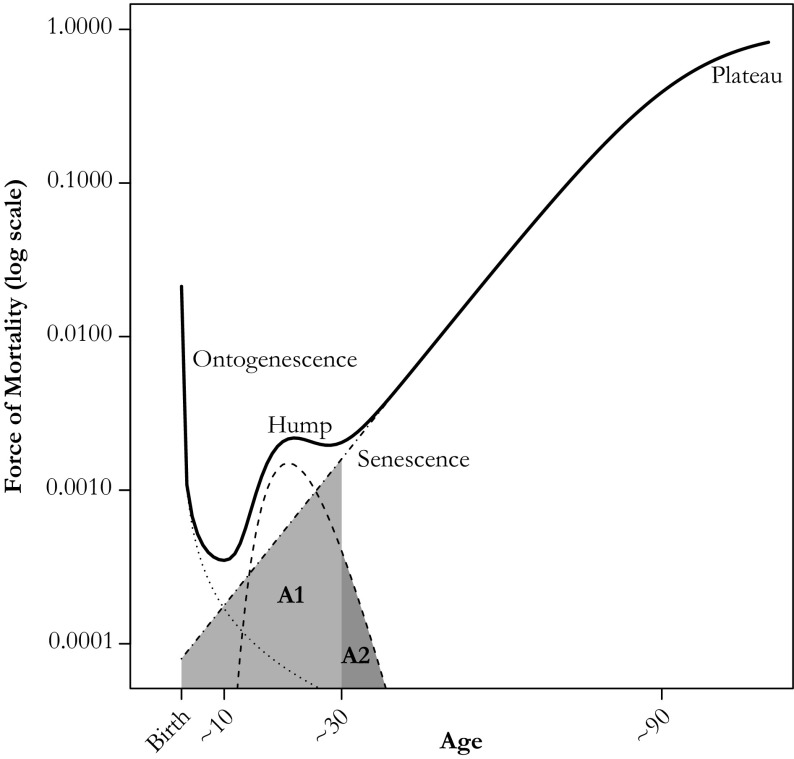
The total force of mortality over the life course is usually composed of three phases: (1) decreasing trend during the first decade of life, (2) hump in the second and third decade, and (3) increasing trend thereafter, marked by a progressive deceleration in very old age. This aggregated evolution does not necessarily reflect the experience of risk in individuals. Area A1 represents senescent mortality between ages 10 and 30, and area A2 represents hump mortality after age 30

Decomposing the shape of mortality entails treating a given mortality age profile as a composite of a set of stylized patterns that capture specific aggregate features of the shape of mortality, and it requires no assumptions about individual risk trajectories. The full age pattern of the force of mortality can be parsimoniously captured by partitioning into three primary phases that may overlap and reflect different biological and social forces (Fig. 1). The first phase, ontogenescence, consists in rapidly declining mortality from birth, possibly because of a combination of “early winnowing of the frailest individuals, acquisition of robustness by the survivors and early concentration of dangerous transitions” (Levitis 2011:806). In most of adulthood, the force of mortality increases at a roughly stable relative pace (Gompertz 1825) in a pattern of somatic decline with increasing chronological age, known as *senescence* (Williams and Day 2003), until about age 90, when it appears to decelerate to a plateau (Horiuchi and Wilmoth 1998; Vaupel 1997).

Between childhood and adulthood, the force of mortality often includes what can be described as a hump. This feature is mostly visible between about 10 and 30 years of age, although it may extend further. We define the hump as a positive deviation from the steady pace of senescence. As a deviation from a Gompertz age pattern, the hump is as much a feature of the rate of change over age as it is of an absolute age trajectory. Mortality humps of similar articulation may appear in both high- and low-mortality contexts. A humpless mortality curve may also have higher mortality in the same age range as an observed hump from a lower-mortality life table.

Some deaths in young adulthood should be attributable to a senescent process governed by the same forces shaping senescent mortality in higher ages (see area A1 in Fig. 1). Indeed, senescence has been argued to start at, or even before, birth (Milne 2006). If we accept this possibility, then the young adult senescent pattern may abide by the same Gompertzian laws as older ages, leaving the hump as an identifiable excess.

We make no assumptions about particular phases within the age patterns of individual causes of death, but we would like to know the extent to which specific causes of death contribute to the all-cause hump. Causes of death differ in the extent to which they contribute to the patterns observed in all-cause mortality. Crucially, some causes of death contribute to the hump, and some do not. Causes of death that contribute to the hump often also contribute to senescent mortality. Cause-specific contributions to the hump may also cover slightly different age ranges, and they may shift over time. Further, strictly senescent causes of death often begin in young adult ages. That is, not all causes of death in the age range of a given all-cause mortality hump contribute to the hump, and even those causes that do contribute to the hump may do so only partially. Common age-cause decompositions of mortality differences (e.g., Andreev 1982; Arriaga 1984; Pollard 1982; Pressat 1985) and studies of the leading causes of death in early adulthood that use arbitrary age ranges, such as 10–34 (Heuveline 2002) or 10–24 (Blum 2009; Patton et al. 2009), do not account for these key aspects of the hump.

We therefore propose a decomposition method that takes into account the shape of mortality, intuitively separates the hump from the rest of mortality, and yields a consistent decomposition by age, cause of death, and shape components. We present a formal description of the method, which follows directly from our definition of the hump as excess mortality. We follow with an application to cause of death data in the United States, comparing our decomposition method with the standard age-cause decomposition of life expectancy. Our method yields a well-suited and informative breakdown of the hump into contributions from particular ages and causes of death. Results isolate excess mortality associated with the transition to adulthood, which would otherwise remain invisible and may be useful to inform theory and policy relating to vulnerability in this phase of the life course.

## Methods

In general, *excess mortality* can be defined as all deaths that exceed what one would expect from a reference pattern of mortality. A reference may be a mortality profile from a different time point, another population, or a subpopulation. The reference we use to measure the hump is that set by the prevailing level of ontogenescent and senescent mortality. In this sense, our approach can be seen as a shape-based method of mortality rate decomposition operationalized by defining an additive model in which the force of mortality is the sum of different components corresponding to the phases described. Each component describes a particular simplified mortality pattern that is more or less expressed during a specific period of the life course: namely, ontogenescence, the hump, and senescence, which may include a plateau at very old ages.

Figure 1 illustrates this additive construction and hints at the arbitrariness of setting strict age bounds for the hump. In this example, the total force of mortality starts increasing again around age 30. Setting age 30 as the end of early adulthood, however, would result in attributing senescent deaths before age 30 (area A1, Fig. 1) to the hump and ignoring deaths after age 30 that belong to the hump component (area A2). Ignoring the overlapping structure of mortality phases or components overlooks the possibility that a given death could be due to any of these forces, especially in young adult ages.

The method we propose combines two common tools of demographic analysis: competing hazard models and cause deletion. We combine these approaches by deleting each cause of death and observing the change in the shape components, which can be interpreted as the contribution of each cause to each component. A similar idea was used in the past to split cause-of-death contributions into ontogenescence and senescence (Gage 1991), but this approach used the parametric model of Siler (1979), which omits the hump. Instead, we use a nonparametric approach in all steps of our method. To preserve coherence between all-cause mortality and cause-specific partitions, the estimation of cause-specific contributions is simultaneous and constrained.

To simplify fitting, we work with mortality rates truncated at the age of observed minimum mortality (near age 10) and 90, which are overwhelmingly attributable to the hump and senescence. Because components are estimated nonparametrically, the senescence component is capable of accommodating a plateau in very old ages if appropriate. Therefore, only hump and senescent components are fit between ages 10 and 90. Formally, the method involves three steps:

1. Reduce the set of causes of death to just those that are potential contributors to the young adult hump.
2. Estimate the senescent and hump components of all-cause mortality.
3. Reestimate both components on cause-deleted data sets, interpreting reductions in the hump component as cause-specific contributions to the hump.

**Fig. 2:**
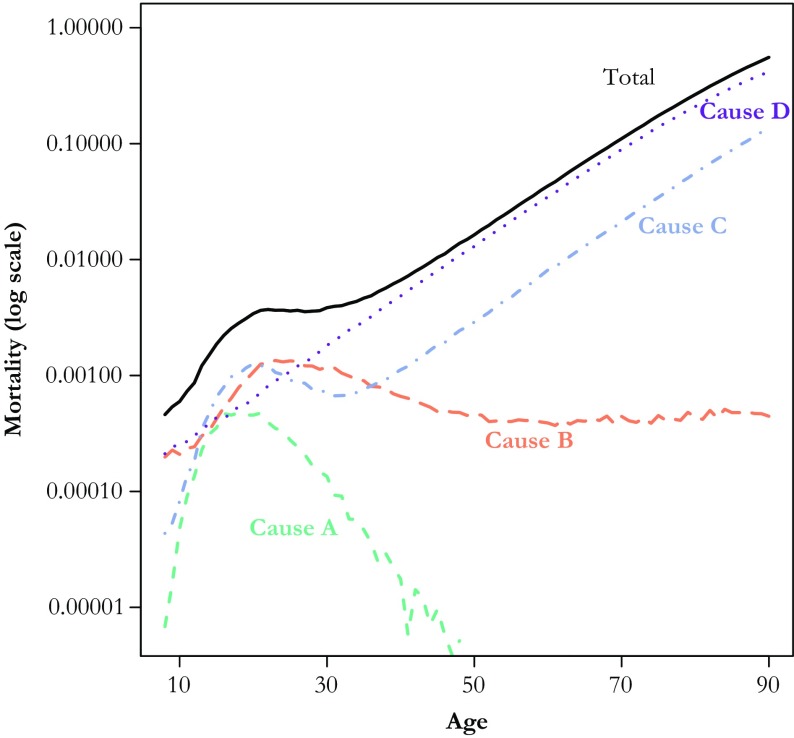
Cause- and age-specific death rates for simulated example. Causes A, B, and C contribute to the hump

The first two steps are essentially applications of existing techniques; the innovation rests in the last step. Herein, we illustrate each step with the same toy example (Fig. 2) involving, for ease of presentation, only four causes of death: A, B, C, and D. Cause A displays a marked hump between approximately age 15 and age 40, thereafter decreasing to a very low intensity. Cause B also displays a hump, albeit higher and much wider, with stable levels after age 40. Cause C combines a hump and a Gompertz trend, making it the leading cause of death between ages 13 and 20, before dropping below Cause B until approximately age 40, when the Gompertz pattern dominates. Cause D does not display any hump and follows a Gompertz trend from age 10 onward. Cause D is the leading cause of death after age 27 and is constantly above Cause A, even at the peak of its hump.

In terms of absolute mortality levels (e.g., death counts), if we define early adulthood as ages 10–34, these causes of death rank as follows: D > B > C > A. The contributions of these causes to the deviation in the force of mortality (the hump), however, are in fact quite far from this. In particular, the shape of the age- and cause-specific death rates shows that Cause D does not contribute at all to the deviation in the force of mortality because it does not display any form of deviation around these ages. The method that we propose takes into account the contribution to the *deviation* of the force of mortality, rather than its absolute level.

### Identify Contributing Causes of Death

The first step in the decomposition of cause- and age-specific contributions to the young adult mortality hump is to identify the causes that are potential contributors to the hump. This step facilitates both the estimation and the interpretation of results. This selection can be based on theoretical arguments or, alternatively, can follow a more inductive approach. The latter is especially useful in the case of many cause-of-death categories, where potential contributors could easily be overseen if cause selection were entirely subjective.

In our application, we use principal component analysis (PCA) to identify the set of causes that potentially contribute to the hump, but other techniques can be used to identify the best candidates (see the section Cause-of-Death Selection). In general, such data-driven techniques allow an exhaustive exploration of data sets containing large numbers of causes of death. In practice, though, manual rearranging of the cause-of-death typology is often advisable. Including too many causes often results in some contributions being too small to be estimated, which causes convergence issues in fitting the decomposition model. Selecting too few causes limits the depth of the analysis. From our toy example, it is clear without any statistical criterion that Causes A, B, and C should be included and Cause D should be excluded.

### Estimate All-Cause Components

In the second step, we estimate additive shape components on the all-cause force of mortality by fitting multiple-component models, sometimes known as *competing hazard models* (Gage 1993), which decompose the force of mortality into additive components that reflect specific patterns in different age ranges. Historically, these models have often been defined by parametric functions (for examples that include a young adult mortality hump, see Heligman and Pollard 1980; Kostaki 1992; Mode and Busby 1982; Thiele 1871). Parametric models are, however, limited because they sometimes fail to adapt to the diversity of mortality schedules, and they have been criticized for the high correlation between their parameters (Dellaportas et al. 2001). This lack of flexibility is particularly crucial when dealing with cause-specific and cause-deleted mortality patterns, which cannot be easily described by fixed mathematical laws. These limitations motivated the development of a nonparametric alternative based on *P*-splines, called the *sum of smooth exponentials* (SSE) model (Camarda et al. 2016).

The SSE model, in its original mortality application, describes the force of mortality over age as the sum of three components that are similar in their interpretations to the ones defined by Heligman and Pollard (1980). In our case, we limit the ages to 10–90 and fit only two of these components, for the hump and senescence. Thus, the force of mortality, **μ**, is modeled as the sum of two vectors **γ** = [**γ**_*H*_: **γ**_*S*_] over *m* ages. The subscripts denote which mortality component each **γ**_*j*_ refers to: hump and senescence, respectively. The model assumes that observed deaths **d** are realizations from a Poisson distribution with a composed mean:

1 $$ \mathbf{d}\sim P\left(\mathbf{e}\boldsymbol{\upmu } =\mathbf{C}\boldsymbol{\upgamma } \right), $$

where **e** is the population under exposure, and **C** is given by

2 $$ \mathbf{C}=\left[\mathbf{E}:\mathbf{E}\right], $$

where **E** = diag(**e**) is the diagonal matrix of the exposure population.

In this way, the composite matrix **C** is an *m* × 2*m* matrix containing the population exposures in duplicate. The role of **C** is to multiply each component by the exposures and simultaneously sum them to obtain the expected values in Eq. (1). The model thus takes the form of a composite link model (Thompson and Baker 1981) and is estimated with a penalized reweighted least squares algorithm (Eilers 2007).

Unlike parametric models, the SSE model does not require strong assumptions about the functional form of each component. For each component, we assume a discrete sequence, and we apply the exponential function to ensure nonnegative elements:

3 $$ {\boldsymbol{\upgamma}}_j=\exp \left({\mathbf{X}}_j{\boldsymbol{\upbeta}}_j\right),\kern0.5em j\in \left\{H,S\right\}. $$

In other words, each component is described by a linear combination of a model matrix **X**_*j*_ and associated coefficients **β**_*j*_. The design matrices **X**_*j*_ can represent parametric or, as in our case, nonparametric structures, such as equally spaced *B*-splines. In this way, the composite force of mortality **μ** can be viewed as a sum of two exponential components, which potentially can be smooth. Further, the SSE model allows us to incorporate shape constraints to enforce senescence and young adult components to be monotonically increasing and log-concave, respectively. These minimal constraints guarantee the identifiability of the model by ensuring that the two components are not interchangeable.

**Fig. 3:**
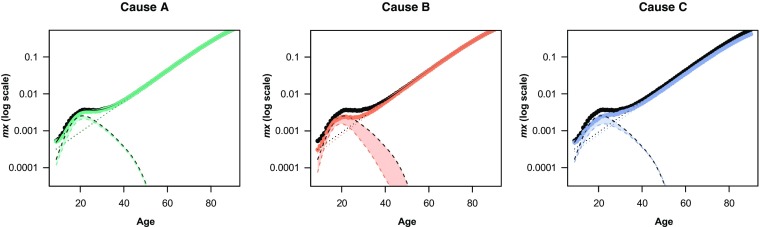
Cause-deleted mortality for simulated example. All-cause and cause-deleted mortality are plotted in black and color (visible in the online version of the article), respectively. The fitted force of mortality, hump and senescence components are plotted in solid, dashed, and dotted lines, respectively. The difference between all-cause and cause-deleted hump components (shaded areas) represents the cause-specific contribution to the hump component

By fitting an SSE model to the overall force of mortality (Fig. 3, black lines), we distinguish the expected deaths that are due to hump mortality $\left({\widehat{\mathbf{d}}}_H=\mathbf{e}{\widehat{\boldsymbol{\upgamma}}}_H\right)$ from those that are due to senescence $\left({\widehat{\mathbf{d}}}_S=\mathbf{e}{\widehat{\boldsymbol{\upgamma}}}_S\right)$. This additive construction acknowledges that deaths that occur during early adulthood are specific not only to this phase of the life course but also partly to the prevailing pattern of senescence.

### Cause-of-Death Decomposition

Building on the SSE model, we propose a constrained approach to decompose the estimated hump into cause- and age-specific contributions $\left({\boldsymbol{\updelta}}_H^{\upkappa}\right)$. The cause-specific contributions to each component *j* can be defined as the difference between a given component estimated from all-cause mortality and the same component estimated on cause-deleted data: ${\boldsymbol{\updelta}}_j^{\upkappa}={\boldsymbol{\upgamma}}_j-{\boldsymbol{\upgamma}}_j^{-\upkappa}$, where κ indicates the cause of death among those previously identified.

Both $\left({\boldsymbol{\updelta}}_H^{\upkappa}\right)$ and $\left({\boldsymbol{\updelta}}_S^{\upkappa}\right)$ for each of the two components (**γ**_*H*_ and **γ**_*S*_) are estimated by refitting simultaneously the SSE model on cause-deleted data. In our simulated example, this step involves only three causes: A, B, and C. This constrained model can then be written as a system of constrained SSE models, such as

4 $$ \left\{\begin{array}{l}{\mathbf{d}}^{-A}\sim P\left({\mathbf{C}\boldsymbol{\upgamma}}^{-A}\right)\\ {}{\mathbf{d}}^{-B}\sim P\left({\mathbf{C}\boldsymbol{\upgamma}}^{-B}\right)\kern0.5em \mathrm{subject}\ \mathrm{to}\begin{array}{l}\kern1.75em {\widehat{\mathbf{d}}}_H=\mathbf{e}\cdot \left({\boldsymbol{\updelta}}_H^A+{\boldsymbol{\updelta}}_H^B+{\boldsymbol{\updelta}}_H^C\right)\\ {}{\widehat{\mathbf{d}}}_s-{\mathbf{d}}^D=\mathbf{e}\cdot \left({\boldsymbol{\updelta}}_S^A+{\boldsymbol{\updelta}}_S^B+{\boldsymbol{\updelta}}_S^C\right).\end{array}\ \\ {}{\mathbf{d}}^{-C}\sim P\left({\mathbf{C}\boldsymbol{\upgamma}}^{-C}\right)\end{array}\right. $$

The first three expressions define the two components (**γ**^−κ^) of the SSE model as in Eq. (1) but on the cause-deleted death counts (**d**^−κ^). The two constraints in the right part of Eq. (4) ensure that cause-specific contributions sum to the all-cause hump and senescence mortality components, respectively. Actual deaths from Cause D, which does not display young adult excess mortality, are subtracted from the overall estimated senescent deaths to ensure that senescence components from Causes A, B, and C are coherently estimated.

Instead of achieving this optimization subject to equality constraints by Lagrange multipliers or quadratic programming, we adapt the simpler but accurate data augmentation method (van Dyk and Meng 2001). On one hand, the regression problem is augmented by incorporating the equations associated with the constraints. On the other hand, we assign to these equations much larger regression weights. Whereas the equations associated with the first three expressions in Eq. (4) are assigned unit weights, the equations involving the constraints receive a series of weights equal to 10^5^. In this way, we simultaneously estimate and constrain our outcomes.

To do so, we rewrite the constraints in Eq. (4) as functions of the unknowns in the associated system of equations: ${\boldsymbol{\upgamma}}^{-A}=\left[{\boldsymbol{\upgamma}}_H^{-A}:{\boldsymbol{\upgamma}}_S^{-A}\right]$, ${\boldsymbol{\upgamma}}^{-B}=\left[{\boldsymbol{\upgamma}}_H^{-B}:{\boldsymbol{\upgamma}}_S^{-B}\right]$, and ${\boldsymbol{\upgamma}}^{-C}=\left[{\boldsymbol{\upgamma}}_H^{-C}:{\boldsymbol{\upgamma}}_S^{-C}\right]$. For the first constraints involving the hump, we have

5 $$ {\displaystyle \begin{array}{l}{\widehat{\mathbf{d}}}_H=\mathbf{e}\left({\widehat{\boldsymbol{\upgamma}}}_H-{\boldsymbol{\upgamma}}_H^{-A}+{\widehat{\boldsymbol{\upgamma}}}_H-{\boldsymbol{\upgamma}}_H^{-B}+{\widehat{\boldsymbol{\upgamma}}}_H-{\boldsymbol{\upgamma}}_H^{-C}\right)\\ {}\kern1.25em =3\mathbf{e}{\widehat{\boldsymbol{\upgamma}}}_H-\mathbf{e}\left({\boldsymbol{\upgamma}}_H^{-A}+{\boldsymbol{\upgamma}}_H^{-B}+{\boldsymbol{\upgamma}}_H^{-C}\right)\\ {}\kern1.25em =3{\widehat{\mathbf{d}}}_H-\mathbf{e}\left({\boldsymbol{\upgamma}}_H^{-A}+{\boldsymbol{\upgamma}}_H^{-B}+{\boldsymbol{\upgamma}}_H^{-C}\right)\\ {}2{\widehat{\mathbf{d}}}_H=\mathbf{e}\left({\boldsymbol{\upgamma}}_H^{-A}+{\boldsymbol{\upgamma}}_H^{-B}+{\boldsymbol{\upgamma}}_H^{-C}\right).\end{array}} $$

The following equations are associated with the constraints on the senescence component:

6 $$ {\displaystyle \begin{array}{l}{\widehat{\mathbf{d}}}_s-{\mathbf{d}}^D=\mathbf{e}\left({\widehat{\boldsymbol{\upgamma}}}_S-{\boldsymbol{\upgamma}}_S^{-A}+{\widehat{\boldsymbol{\upgamma}}}_S-{\boldsymbol{\upgamma}}_S^{-B}+{\widehat{\boldsymbol{\upgamma}}}_S-{\boldsymbol{\upgamma}}_S^{-C}\right)\\ {}\kern3em =3\mathbf{e}{\widehat{\boldsymbol{\upgamma}}}_s-\mathbf{e}\left({\boldsymbol{\upgamma}}_S^{-A}+{\boldsymbol{\upgamma}}_S^{-B}+{\boldsymbol{\upgamma}}_S^{-C}\right)\\ {}\kern3em =3{\widehat{\mathbf{d}}}_s-\mathbf{e}\left({\boldsymbol{\upgamma}}_S^{-A}+{\boldsymbol{\upgamma}}_S^{-B}+{\boldsymbol{\upgamma}}_S^{-C}\right)\\ {}2{\widehat{\mathbf{d}}}_s+{\mathbf{d}}^D=\mathbf{e}\left({\boldsymbol{\upgamma}}_S^{-A}+{\boldsymbol{\upgamma}}_S^{-B}+{\boldsymbol{\upgamma}}_S^{-C}\right).\end{array}} $$

The factor 2 on the left side of Eqs. (5) and (6) depends on the number of hump-related causes and will always be 1 less than the number of selected causes.

We can now unify both the systems of equations and constraints in Eq. (4) in a single framework. Let $\overset{\smile }{\mathbf{d}}$ and $\overset{\smile }{\boldsymbol{\upgamma}}$ denote the following vectors of response and unknowns:

7 $$ {\displaystyle \begin{array}{l}\overset{\smile }{\mathbf{d}}=\left({\mathbf{d}}^{-A},{\mathbf{d}}^{-B},{\mathbf{d}}^{-C},2{\widehat{\mathbf{d}}}_H,2{\widehat{\mathbf{d}}}_S,{\mathbf{d}}^D\right)\\ {}\overset{\smile }{\boldsymbol{\upgamma}}=\left({\boldsymbol{\upgamma}}_H^{-A},{\boldsymbol{\upgamma}}_H^{-B},{\boldsymbol{\upgamma}}_H^{-C},{\boldsymbol{\upgamma}}_S^{-A},{\boldsymbol{\upgamma}}_S^{-B},{\boldsymbol{\upgamma}}_S^{-C}\right).\end{array}} $$

The proposed approach becomes a single model with a composed mean as in Eq. (1):

8 $$ \overset{\smile }{\mathbf{d}}\sim P\left(\overset{\smile }{\mathbf{C}}\overset{\smile }{\boldsymbol{\upgamma}}\right), $$

where the composite matrix takes the following form:9where **0** represents an *m* × *m* square matrix of zeros. The repetition of the exposure matrices **E** three times in the upper (as block-diagonal) and lower (in block rows) parts of $\overset{\smile }{\mathbf{C}}$ is due to the number of hump-related causes (A, B, and C). The 2 × 2 block structure of $\overset{\smile }{\mathbf{C}}$ is the result of working with an SSE with two components (*H* and *S*).

By augmenting both **C** to $\overset{\smile }{\mathbf{C}}$ and **γ** to $\overset{\smile }{\boldsymbol{\upgamma}}$, we can still write the model as a composite link model, which allows us to estimate the complex decomposition using reliable algorithms to estimate cause-specific contributions to each component while constrained to sum to the overall components. As we demonstrate in the Application section, more hump-related causes can be incorporated by further augmentation of the model elements. An implementation of these methods is available in the MortHump R package on the CRAN repository (Remund et al. 2018).

Figure 3 illustrates the application of this technique to our toy example. The black solid line represents the all-cause age-specific fitted rates, and the black dashed and dotted lines represent the estimated hump and senescence components, respectively. Each graph shows how these components are affected by the deletion of Cause A, B, and C, respectively, and the shaded area illustrates the contribution to the total hump from each cause $\left({\boldsymbol{\updelta}}_H^{\upkappa}\right)$. This figure also helps to characterize the respective contributions of each cause to the shape and size of the hump: (1) the deletion of Cause A affects only the hump component and not the senescence component, whereas the deletion of Causes B and C affects both; (2) the drop in the hump is larger after deletion of Causes B and C, which indicates a larger contribution of these causes to the hump than Cause A; and (3) the decrease in the hump is larger before the peak in Cause C and after the peak in Cause B, which means that their contributions are not centered on the same age.

These characteristics can be better estimated by designing summary measures of the cause-specific contributions to the hump. By working in a smooth setting, we can evaluate the components with fine age granularity, which allows us to consider components as continuous functions $\left({\boldsymbol{\updelta}}_H^{\upkappa}(x)\approx {\boldsymbol{\updelta}}_H^{\upkappa}\right)$. These densities can be used to quantify the hump and its cause-specific contributions.

Although many dimensions of the hump can be studied—such as its height, location, or spread—we focus here on its general magnitude as measured by the potential gain in life expectancy that would result from the deletion of the cause-specific contribution to the hump. The total years of life expectancy lost to the hump can be decomposed by age and cause using standard decomposition techniques (Arriaga 1984). This measure differs from what would be obtained with the direct application of these standard methods because the contribution from the hump represents only a partial reduction of the observed rates rather than a complete elimination of the observed rates in an age range.

In our example, the deletion of the overall hump would generate an increase of 1.65 years of life expectancy, of which 0.3 years (18.2 %) is due to Cause A, 0.78 years (47.3 %) is due to Cause B, and 0.55 years (33.3 %) is due to Cause C. These proportions are very different from the gains in life expectancy induced by the total deletion of deaths between ages 10 and 34 (0.96 years), of which Causes A, B, C, and D would contribute 0.12 years (12 %), 0.3 years (31 %), 0.3 years (31 %), and 0.25 years (26 %), respectively. By taking into account the presence of senescence at these ages and considering only the deaths in the young adult mortality hump, the contribution of Causes A, B, and C to the hump is thus strongly reevaluated.

## Application

### Data

We use an early release of data produced by the Human Mortality Database (2017) on cause- and age-specific death rates for the United States between 1959 and 2015, covering ICD versions 7–10. These data are aggregated from National Center for Health Statistics deaths microdata into 92 cause categories. We first graduate the cause-specific death rates from abridged age groups to single ages using *P*-splines implemented in the MortalitySmooth R package (Camarda 2012); we then constrain them to sum to single-age all-cause mortality rates from the Human Mortality Database. We make no adjustments to smooth potential coding ruptures, but none of the three ICD revisions (in 1968, 1979, and 1999) generate a visible rupture in the patterns we report, except the introduction of the HIV code in 1987.

## Cause-of-Death Selection

From the original 92 cause-of-death codes, we identify those that display a particular shape during early adulthood and are therefore good candidates to contribute to the hump. The causes that are the most susceptible to contribute to the hump are those that display in their shape a high level of change (both positive and negative) during young adulthood. We capture this shape by computing the first difference over age of the all-cause and each cause-specific force of mortality between ages 10 and 34. We then estimate the proximity of each cause’s shape to the all-cause shape by computing the age-wise Euclidean distance. Algebraically,

10 $$ {\Delta}_j^{\upkappa}=\sqrt{\sum \limits_x{\left({\boldsymbol{\uprho}}_{xj}^K-{\boldsymbol{\uprho}}_{xj}\right)}^2,} $$

where ${\Delta}_j^{\upkappa}$ is the Euclidean distance over age *x* for year *j* and cause κ, and **ρ**_*x*_ and ${\boldsymbol{\uprho}}_x^{\upkappa}$ are the first difference over age of the all-cause and cause-specific forces of mortality, respectively. Repeating this analysis for each year yields 52 observations for each cause (1959 to 2015), which can be reduced to two dimensions using PCA.

**Fig. 4:**
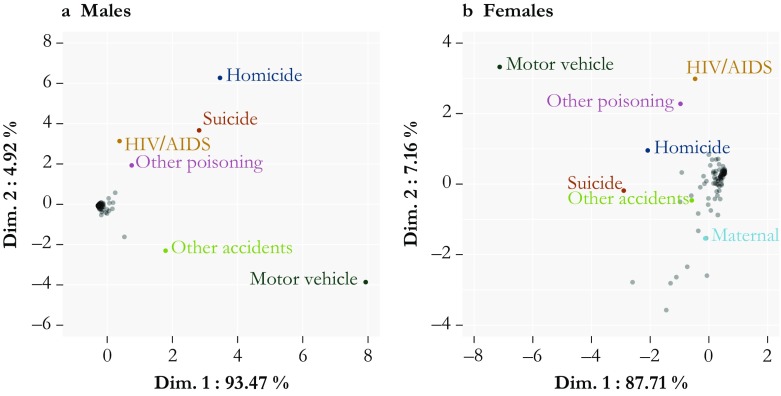
Difference in shape between each cause of death and all-cause mortality from ages 10 to 34. For each year from 1959 to 2015, we compute the first differences of the cause-specific forces of mortality and compare each with the all-cause equivalent using the Euclidean distance as a unique summary measure. The information from these 52 years is reduced by PCA and represented on standardized scales. Seven causes of death are flagged for their unusual shape: traffic accidents, homicides, suicides, poisoning, HIV/AIDS, other accidents, and maternal mortality

As indicated in Fig. 4, the first two axes summarize more than 95 % of the information for both sexes. Thus, the causes that stand out are generally the same over the whole period. The positions of causes on the first two dimensions highlight seven causes that deviate from the rest: motor vehicle accidents, suicides, homicides, other accidents, “other” poisoning (i.e., nonalcoholic, mainly drug overdoses), and HIV/AIDS, as well as maternal mortality for females. The pattern is clearer for males than for females, notably for nontraffic accidents, but we chose to use the same list for both sexes in order to ensure a direct comparison between sexes. We include maternal deaths in our typology because despite its similarity to the other causes between ages 10 and 34, it stands apart at younger ages before converging with the other causes.^1^ This selection of causes is by no means canonical, but it is roughly coherent, and it accounts for the vast majority of the hump.

### Results

#### Magnitude of the Hump and Comparison With Standard Decomposition Methods

**Fig. 5:**
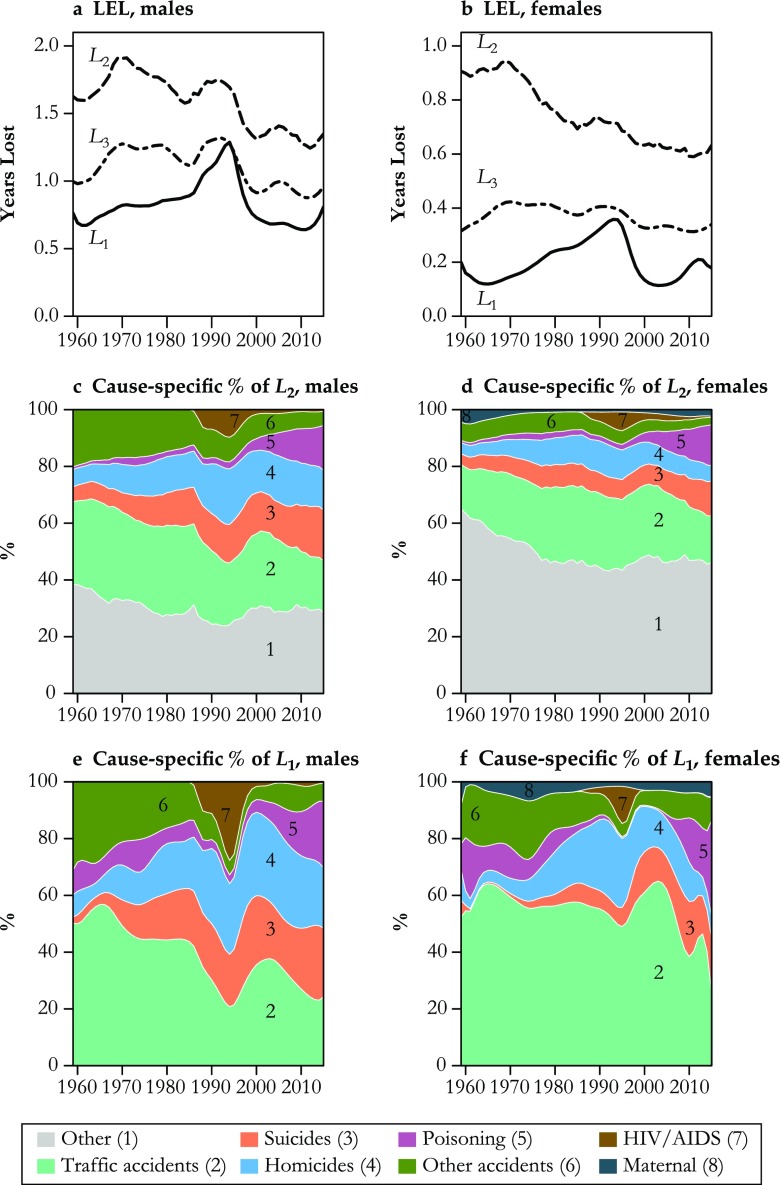
Application of our method to U.S. mortality by sex between 1959 and 2015. The top panel displays the absolute loss in life expectancy between the observed force of mortality and after deleting only the hump component (*L*_1_), all deaths from ages 10 to 34 (*L*_2_), and only those from the previously identified seven causes (*L*_3_). The bottom four panels indicate the cause-of-death shares of *L*_1_ and *L*_2_

Most studies on young adult mortality use absolute age-specific death rates as the basis for measurement and comparison. To demonstrate the difference between our method and approaches based on absolute death rates, we first apply our decomposition method to each year independently to separate the hump from the all-cause mortality rate schedule. The hump is itself a rate schedule, which can therefore be translated to years of life expectancy lost (LEL) (Arriaga 1984). For comparison, we translate three total rate schedules to years of LEL (Fig. 5):

- *L*_1_: The hump only $\left({\boldsymbol{\upgamma}}_H={\sum}_{\upkappa}{\boldsymbol{\updelta}}_H^{\upkappa}\right)$
- *L*_2_: All-cause absolute death rates between ages 10 and 34 (_25_*m*_10_)
- *L*_3_: Sum of the seven selected causes, between ages 10 and 34 $\left({\sum}_{\upkappa\ 25}{m}_{10}^{\upkappa}\right)$.

The LEL due to the seven selected causes (*L*_3_) is by definition smaller than that generated by all-cause mortality (*L*_2_) (Fig. 5, panels a and b). Most of the time, hump LEL (*L*_1_) is also lower than these seven causes (*L*_3_) because the senescent component has been removed. In some cases, such as in the early 1990s, a crossover is observed because the hump extends beyond the fixed age range used for the two comparisons (ages 10–34 in our case). The gap between all-cause (*L*_2_) and seven-cause LEL (*L*_3_) decreases over time because of the decreasing share of other causes in deaths occurring between ages 10 and 34.

Although these three series share some similarities, such as an initial increase in the 1960s and a sharp decrease in the late 1990s, trends differ in key ways. Specifically, trends in the hump and all-cause (10–34) LEL are even of opposite sign. Between the early 1970s and the mid-1980s, all-cause absolute LEL decreased by 18 % for males (1971–1984) and 27 % for females (1969–1985), while the hump impact increased by 7 % for males and 87 % for females. Thus, during this period, the decrease in all-cause absolute LEL was due to changes in the senescence component, while the hump continued to grow. We show later that this increase in the hump component comes from its widening rather than an increase in intensity around its peak.

Panels c and d of Fig. 5 show the proportional cause-of-death contributions to LEL from all mortality in ages 10 to 34. The proportional contribution of “other causes” decreases from 40 % to 30 % among males and 65 % to 45 % for females over the whole period. This is consistent with the observation from Fig. 5, panels a and b, that the gap between the all-cause LEL and the seven-cause LEL declines over time. The sudden compositional shift around 1987 is due to the introduction of HIV as a new cause-of-death category.

Figure 5, panels e and f, shows the cause-of-death composition of the hump-only LEL for males and females. Traffic and other accidents made up approximately 80 % of the male hump LEL in the 1960s. This situation, however, slowly evolved over time, and currently these two causes account for only less than one-third of the hump LEL. Meanwhile, suicides and homicides have grown from less than 10 % to approximately one-half of the male hump LEL. The contribution of poisonings grew from less than 5 % to almost one-quarter of the hump LEL in the last two decades. The story for males is thus very much about suicides, homicides, and poisonings replacing traffic accidents in the composition of the hump. In comparison, the pattern for females is more of a stable domination of traffic accidents in hump LEL, with momentary increases from homicides, other accidents, and recently a surge of the share of poisonings.

There are important compositional differences between causes in the hump-only (Fig. 5, panels e and f) versus the cause-specific LEL based on absolute rates (panels c and d). For example, the relative importance of HIV to hump LEL was much higher than absolute death rates would suggest. The portion of years of hump LEL produced by traffic accidents is also higher than that of absolute traffic accident death rates in all years. Moreover, the impact of homicides is stronger on the hump than on absolute rates. The ranking of causes contributing to LEL is also different when we separate the hump versus absolute death rates at ages 10–34, even ignoring “other causes.” On average, the mean number of differences in ranking among causes between the two methods is 1.47 for males and 4.25 for females. There are even years (1993 to 1996 for females) for which none of the seven causes occupies the same rank in both methods.

#### Shape of the Hump by Cause, Over Age, Time, and Cohorts

**Fig. 6:**
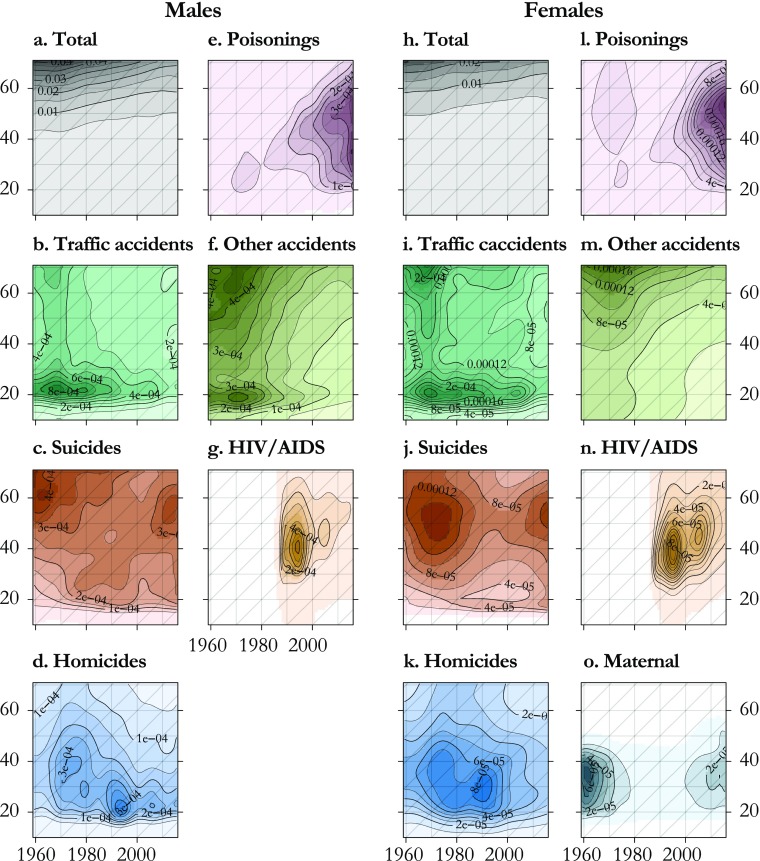
Lexis surfaces of cause-specific death rates, U.S. males and females 1959–2015. Each cause is plotted on a dedicated color scale (visible in the online version of the article), and contours are superimposed to give an indication of the magnitude

Results on the magnitude of the hump omit the shape of cause-of-death contributions to the hump. We visualize patterns on the rate scale by plotting smoothed values over age and time on Lexis surfaces. Figure 6 shows surfaces of raw (undecomposed) cause-of-death rates. Visually, the all-cause surface does not reveal any hump because all rates for young ages are dwarfed by the levels reached in old age as a result of the senescence component. This is also the case for nontraffic accidents, which have a strong senescence component. All other causes of death that were identified as potential contributors to the hump display relatively high mortality during early adulthood. Some causes, however, combine this with other age patterns; for example, traffic accidents present a bimodal shape, with high mortality levels during early adulthood as well as old age (not visible because of truncation of ages above 70). Suicides also strongly affect older males and middle-aged females. This picture of raw rates confirms that the causes of death that contribute to the hump often also contribute to senescence or ontogenescence. Considering all deaths from these seven causes as relating to the young adult mortality hump would be an overgeneralization.

**Fig. 7:**
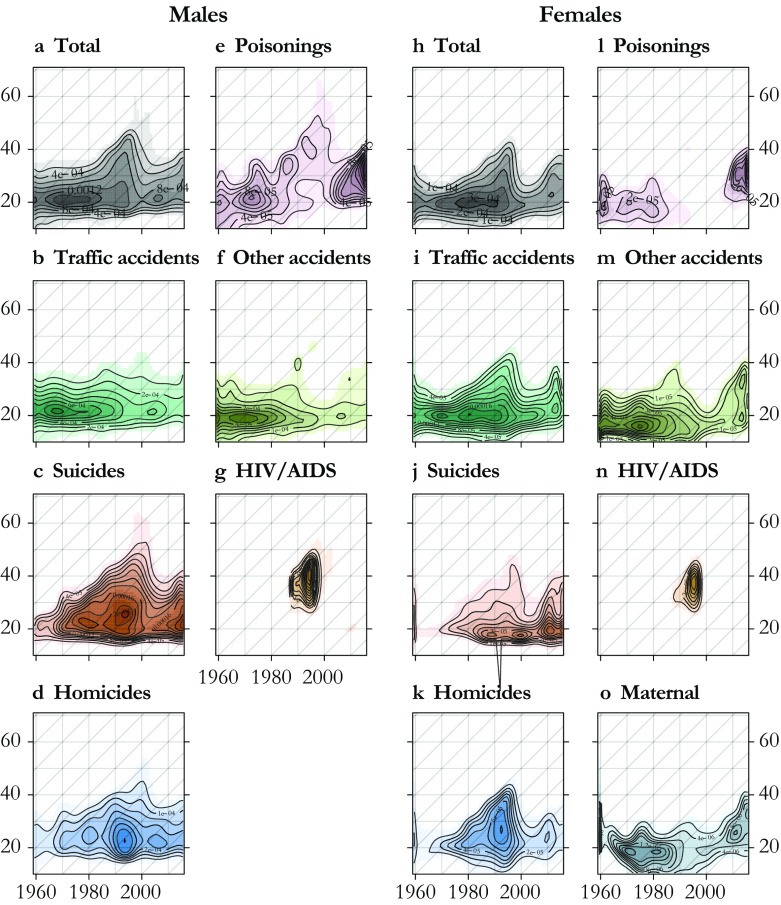
Lexis surfaces of cause-specific contributions to the hump, U.S. males and females 1959–2015. These correspond to the ${\delta}_1^{\kappa }$ computed for each year. Each cause is plotted on a dedicated color scale (visible in the online version of the article), and contours are superimposed to give an indication of the magnitude

We apply our model to the mortality of U.S. males and females from 1959 to 2015 using the same set of causes of death. The cause and age-specific contributions are presented in the form of smooth Lexis surfaces (Fig. 7). In these surfaces, the hump is now clearly visible, having been separated from the senescence component. The all-cause hump is stable in neither intensity nor age range over time (Fig. 7, panels a and h). From the beginning of our study period until the 1980s for males and 1970s for females, the hump is relatively compact and centered on age 20. The male hump is generally higher and wider than the female hump. Maximum age-specific hump contributions come from approximately age 20 in the 1970s for males and from approximately age 20 in 1980 for females. The hump then widens progressively into the 30s and 40s, until approximately 1997, when it suddenly shrinks. Since 2000, the hump has resumed a process of widening into the 30s for both males and females.

Because these peculiarities in all-cause hump patterns are the sum of contributions from different causes of death, the primary contours in the all-cause hump are best explained in terms of its cause components. It is convenient to describe patterns in the contributions of each cause of death in terms of age, period, and cohort patterns. *Age patterns* here refer to sudden increases or decreases in a contribution to the hump in a narrow age range over a wide range of years, and these are visible in the form of horizontal contours in the surfaces. *Period patterns* refer to simultaneous changes in contributions to the hump over a broad range of ages, producing vertical contours in the surfaces. *Cohort patterns* here refer to differences in contributions to the hump between adjacent birth cohorts, producing contours running in 45-degree diagonal lines. Each pattern is clearly visible in at least some of the causes contributing to the hump.

Age patterns are to some extent visible in each cause of death contributing to the hump. In the all-cause hump, this age effect manifests itself with a rapid increase hump mortality between ages 15 and 20, and a narrow peak between ages 20 and 25. The age patterns in increase and the peak are found in each of the hump causes of death except poisoning for males (Fig. 7, panel e). For HIV/AIDS, onset follows a similar age pattern, but it happens later, at approximately age 30 (panels g and n). Patterns in the decline of the hump in higher ages are far less regular over time and causes of death and show few clear horizontal contours, except perhaps traffic and other accidents among males (panels b and f).

Period patterns in cause contributions to the hump are associated with the emergence of new threats that hit young adults particularly hard, or with new technologies or policies that simultaneously reduce risk over a range of young adult ages. The all-cause hump shows a strong period pattern in the form of a sudden decrease for both males and females around 1997. This pattern is mostly accounted for by HIV/AIDS for males (Fig. 7, panel g) but coincides with simultaneous drops in homicides for females (panel k). A smaller period decrease is visible for males in the early 2000s, caused by simultaneous decreases in the hump contributions of suicides, homicides, and poisonings. Period increases are visible for HIV/AIDS for both males and females starting in the late 1980s (panels g and n)^2^ but also for homicides in the early 1990s (panels d and k).

Cohort patterns are primarily visible in the upper edge of the hump—that is, in the way the hump fades into senescence. We see this pattern in male poisonings and suicides (Fig. 7, panel e and c) and in female homicides and traffic accidents (panels i and k), each starting in cohorts born around 1950. Most cases of cohort hump effects in these results are paired with a constant age at onset, leading not only to an extension of the hump into higher ages but also to a general widening of the hump. Male poisonings (panel e) are an exception to this given that this cause also displays a shift in age at onset.^3^

### Discussion of Application to U.S. Data

*Age patterns* concern the age at onset, peak, and fading of the hump into senescence. The former is relatively stable between ages 10 and 15 and applies to all causes of death except poisoning for males. This stability suggests that this dimension of the hump could be expressing a form of turmoil inherent to the nature of adolescence, as often conceived in the psychological literature (Freud 1968; Hall 1904). Recent studies have shown peculiar neurological developments in the adolescent brain,^4^ which, according to some authors, generate a mismatch between the ability to anticipate and the regulation of emotions that could explain why adolescents more often engage in dangerous activities, particularly under peer pressure (Casey et al. 2011; Steinberg 2005).

The strong and regular patterns that we observe in young adult excess mortality risk are consistent with these theories. That the peak in excess mortality occurs five or more years after these neurological changes may indicate a mortality lag between the acquisition of behaviors and mortality, because of either a phase of latency or the cushioning effect of age-related policies, such as legal ages at driving or alcohol consumption. However, if an endogenous biological process were a sufficient explanation for the hump, we would observe only age patterns. Such regularity is apparent only in onset and peak for most (but not all) hump causes. We see irregularity in the location of the tail end of the hump, as well as strong period and cohort patterns for some causes, which point to other nonbiological mechanisms.

The shape of the hump is indeed marked by shocks, both positive and negative, that are specific to certain years or periods of time. A striking example of this pattern is the recent rise in poisoning mortality for both males and females (Fig. 7, panels e and l). Although it affects ages beyond the hump (Fig. 6, panels e and l), this new phenomenon contributes to a strengthening and widening of the hump. This health crisis, mainly due to a sharp increase in opioid overdoses, has attracted scientific and political attention (e.g., Hedegaard et al. 2015).

Homicide contributions to the hump also display period patterns (Fig. 7, panels d and k). A first rapid increase took place in the late 1960s to early 1970s, which particularly concerned young adults (LaFree 1999). A second peak came in the early 1990s, particularly affecting young, black, and Hispanic males, and quickly decreasing after 1993 (Cook and Laub 2002). Our results show that females experienced an effect in the mid-1990s over a wider age range, possibly because of the presence of an accompanying cohort effect or underlying patterns in the age and sex differences between victims and perpetrators. There is no consensus about the causes of this wave of violent criminality. Explanations often involve changes in social support and economic inequalities (Pratt and Godsey 2003), changes in size and strategy of police forces, and changes in crack cocaine markets (Cook and Laub, 2002; LaFree 1999; Levitt 2004).

The strongest example of such period effects is undoubtedly the rapid spread of HIV/AIDS, for which ICD codes began to capture only in 1987 (Fig. 7, panels g and n). This cause of death not only increased the intensity of the hump but also contributed to its spread into higher ages—well into ages 30–40. It suddenly diminished in 1996 with the introduction of antiretroviral therapies, which postponed the age at death beyond the hump (Palmisano and Vella 2011). This drop in HIV/AIDS deaths explains a portion of the strong period effect observed in the all-cause hump between 1995 and 1998 but not entirely given that our results show additional drops for suicides among males (panel c) as well as homicides for females (panel k).

Both the male and female all-cause hump display a clear progressive widening that started around 1980 and 1970, respectively, before it abruptly narrowed in the late 1990s (Fig. 7, panels a and h). This could potentially be interpreted from a period point of view as a progressive extension of the period of young adult excess mortality, but the fact that this widening happens roughly at a regular pace of one year of age per calendar year suggests that this may be a cohort effect concerning people born around or after 1950. This cohort experienced a higher mortality than earlier cohorts and contributed to a widening of the hump by progressively increasing the tail age of the hump.

For suicides and homicides, an age effect is superposed on the cohort effect, generating a triangle pattern on the lexis surfaces (Fig. 7, panels c and d) but not for poisonings (Fig. 7, panel e). When the cohorts born between roughly 1945 and 1970 leave the age range of the hump, their higher mortality for these specific causes is retained but blends into the general level of senescence. This pattern is accompanied by a genuine period decrease in these causes around 1997 (visible in Fig. 6, panels c and d) but could also explain some of the period patterns. For males, this cohort effect is obvious for suicide and poisonings and is more subtle for homicides from the 1960s to the 1990s. This predates the equivalent widening observed on the all-cause hump by about one decade. The male hump appeared to spread later than the female hump because it was initially broader because of a wider contribution of traffic accidents, which temporarily concealed cohort patterns in homicide, suicide, and poisonings that had begun in the 1960s (Fig. 7, panels c–e).

These cohort observations confirm previous findings that mortality increased for the cohorts born after 1945 for suicide (Chauvel et al. 2016; Stockard and O’Brien 2006), homicide (O’Brien and Stockard 2002, 2006), and poisoning (Miech et al. 2011), reaching a peak with cohorts born in the 1960s. This cohort phenomenon has been given similar explanations for each of these three causes of death, including a large relative cohort size (a so-called Easterlin effect), a higher percentage of nonmarital births, higher exposure to drug use, and a lower degree of social integration and regulation (O’Brien and Stockard 2002, 2006; Stockard and O’Brien 2006) as well as the rise of socioeconomic stressors, particularly among non-Hispanic, low-educated, and unmarried members (Chauvel et al. 2016).

The resemblance in the patterns of homicide, suicide, and poisoning is thus partly explained by the fact that they share common underlying social dynamics. There is also a risk of misclassification between all three causes, but particularly between suicide and poisoning. Qualitative studies have suggested that suicides may be classified as poisonings to a significant yet unmeasurable extent (Miech et al. 2013:138). Some of the similarities between the hump contributions from suicide and poisoning may be due to this kind of coding imprecision, but we have no reason to suspect that aggregate patterns are accounted for by coding peculiarities.

## Conclusion

We conceive of the young adult mortality hump as excess mortality beyond the prevailing senescent level of mortality. Research on young adult mortality should consider this difference when focusing on this specific phase of the life course. We propose a method to flexibly measure the hump and decompose it into its cause-of-death contributions. This method characterizes the hump not merely in terms of peak location (Goldstein 2011) or a restrictive set of parameters (e.g., Heligman and Pollard 1980), but it estimates a full schedule of hump mortality rates by cause of death.

We apply this method to mortality rates by cause of death in the United States from 1959 to 2015 and offer a first look at trends in the hump in isolation from background mortality. When isolated, trends in the U.S. hump differ qualitatively from trends in observed mortality rates in the same age range. Specifically, we document countervailing trends in the hump magnitude based on decomposed versus observed rates. When mortality rates are broken down by cause of death, we also observe differences in the magnitude, ranking, and trends in particular cause-of-death contributions to life expectancy lost to the hump. The age of the peak of the hump has been relatively stable in the United States, but its spread has undergone large and regular changes articulated along age, period, and cohort lines.

More specifically, the results show a progressive widening of the hump starting in the 1960s and coming to an abrupt halt in the late 1990s. This pattern is due partly to the excess mortality of the cohorts born after 1950 in suicides, homicides, and poisonings, as well as period shocks, such as the rise and fall of the HIV/AIDS epidemic in the late 1980s and early 1990s. The hump for males in the United States was larger than that for females, and the contribution of traffic and other accidents decreased continuously, which was offset by increases in contributions from suicides, homicides, and poisonings. The female mortality hump was mainly driven by traffic accidents, homicides, and recently poisonings. For both males and females, HIV/AIDS played a much more important role in the hump than it did in overall observed mortality rates.

The application of our method to U.S. data reveals mortality patterns that otherwise remain partially hidden from view and analysis. It is our hope that better measurement will lead to increased understanding of the force of mortality in young adult ages and the relationship between other changes during young adulthood and aggregate mortality outcomes. Such applications could fruitfully target specific subpopulations and contexts. Simultaneous estimation of the model over age and time would help stabilize results for such specific analyses of smaller populations.

## Electronic supplementary material

ESM 1 (PDF 387 kb)

## Footnotes

1 This was suggested by sensitivity analyses. These analyses are not shown here, but we show how to replicate them in Online Resource 1 (Fig. S8).

2 The ICD code for HIV/AIDS was introduced in 1987, although this cause of death existed earlier. However, its spread was fast enough that it can be considered a period shock.

3 When running the same decomposition in the cohort perspective, we see no such cohort pattern on the hump for poisoning among males born in the 1950s (only an excess around age 20), which suggests that this excess mortality is broadly specific to this cohort and not merely an attribute of its young adulthood. Figure 6, panel e, supports this speculation. In the period perspective, this produces an apparent cohort effect in the hump, which eventually dissolves into senescence.

4 These changes concern the dopaminergetic activity due to the unsynchronized development of (1) myelination and gray matter in prefrontal areas of the brain that control social cognition and anticipation, and (2) the limbic system that controls emotions and feelings of reward (Giedd 2004; Lenroot and Giedd 2006; Steinberg 2010).
