# Supplementary material for: A Cause-of-Death Decomposition of Young Adult Excess Mortality
Source: Demography. 2018 Jun 4;55(3):957–78. doi: 10.1007/s13524-018-0680-9 (PMC5992252; doi:10.1007/s13524-018-0680-9)
Supplement: Supplementary file 1 — (PDF 387 kb) [file 13524_2018_680_MOESM1_ESM.pdf]

Online Resource 1

A cause-of-death decomposition of young adult excess mortality

Sensitivity analyses

Adrien Remund[1,2,4], Carlo G. Camarda[2], and Tim Riffe[3]

[1] *University of Geneva, Institute of Demography and Socioeconomics*
[2] *Institut national d'études démographiques*
[3] *Max Planck Institute for Demographic Research*
[4] *Swiss National Centre of Competence in Research LIVES - Overcoming Vulnerability: Life Course Perspectives*

December 13, 2017

Abstract

The aim of this supplementary material is to provide readers with sensitivity analyses on the model of cause-of-death decomposition of the young adult mortality hump. We first explore the consequences of choosing alternative sets of causes that contribute to the hump. We then test the ability of induction via principal components analysis to identify the best typology. We finally show the impact of choosing alternative age intervals for the inductive methods.

Introduction

When presented with a dataset of age- and cause-specific death rates, it may be difficult to intuitively identify which causes of death likely contribute to the hump. In the paper we suggest an inductive approach to select these causes in order to adapt to each context and avoid excluding causes that should have been included. The motivation for reducing the cause of death list to a minimal set that contributes to the hump is primarily one of computational parsimony. In our case we started with 92 causes and ended up with a set of seven. There are $\binom{92}{8}$ = 8+ billion ways that seven causes could have been selected from this initial set, and very many more given that the number seven is itself a flexible set size. This means that there are many ways in which the selection of hump-contributing causes might go awry. For example, a hump contributing cause may erroneously be left out, or non-contributing causes may be uselessly included in the decomposed set. In this supplementary material we design a simulation example in order to (1) confirm the ability of the proposed PCA to identify a good cause set, (2) investigate the potential biases that could be introduced by making a poor choice of causes of death, and (3) explore the effect of choosing alternative age ranges in the PCA.

1 Generating simulated forces of mortality

We start by generating eight cause-specific forces of mortality, using a parametric model inspired by Kostaki (1992), but without an ontogenescence component and with a stronger leveling off at older ages. This is to show how the flexibility of the non-parametrical approach can fit even shapes that do not strictly follow a Gompertz trend. The force of mortality $\mu(x)$ is described by the following formula,

$$\mu(x) = \begin{cases} d \cdot \exp(-e \cdot (log(x) - log(f))^2) + \frac{g \cdot h^x}{1+4g \cdot h^x} & \forall x \leq f \\ \\ d \cdot \exp(-e/k \cdot (log(x) - log(f))^2) + \frac{g \cdot h^x}{1+4g \cdot h^x} & \forall x > f \end{cases} \quad (1)$$

The first term captures the young adult mortality hump and is defined by parameters d (height), e (spread), f (location) and k (asymmetry). The second term captures the exponential increase of the risk of death associated with senescence, including a leveling off at very old ages, and is defined by parameters g (level) and h (slope). For our simulation exercise, we define eight causes of death (A to H), with different parameters (d to h). The parameters used for the simulation are presented in Table S1.

		Cause (κ)							
		A	B	C	D	E	F	G	H
Paramter	d	1.5e-03	0.00135	0.00105	0	0	0	0	0
	e	8	8	8	8	8	8	8	8
	f	18	24	20	20	20	20	20	20
	k	0.7	0.5	1	1	1	1	1	1
	g	5.0e-06	2e-40	1e-5	3.750e-06	1.875e-06	1.875e-06	1.875e-06	3.75e-07
	h	9.8e-01	1.01	1.105	1.105	1.12	1.05	1.15	1.17

Table S1: Parameters used for the simulated cause-specific forces of mortality

Figure S1 displays the simulated forces of mortality computed from these parameters, from 8 to 90 years of age. Causes A, B and C include a hump component, while causes D through H don't. Cause A almost only consists of a hump, with very low levels of senescence, and resembles what is often observed for homicides or traffic accidents. Cause B displays a sharp increase in the late teens but levels off thereafter, as is often observed with suicides. Cause C combines a hump and a senescence component, resembling what is often observed with falls or non-traffic accidents. The other causes display no hump but have varying initial levels (g) and rates of ageing (h), including also a progressive leveling off in old age.

The dashed black line in Figure S1 represents the true all-cause hump, which is the sum of all cause-specific ($\kappa \in A...H$) hump terms: $\gamma_H = \sum_\kappa \gamma_H^\kappa$, where

$$\gamma_H^\kappa(x) = \begin{cases} d^\kappa \cdot \exp(-e^\kappa \cdot (log(x) - log(f^\kappa))^2) & \forall x \leq f^\kappa \\ d^\kappa \cdot \exp(-e^\kappa / k^\kappa \cdot (log(x) - log(f^\kappa))^2) & \forall x > f^\kappa \end{cases} \quad (2)$$

.

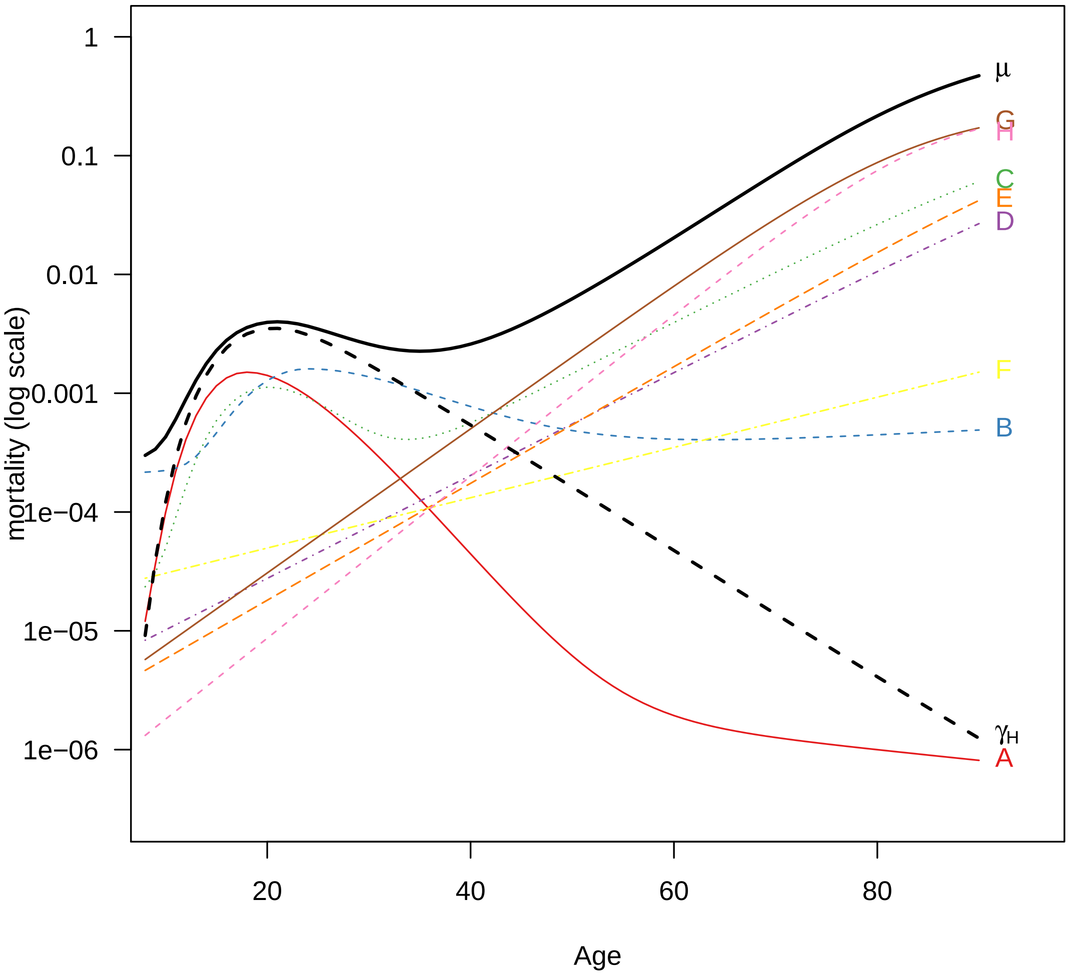

Figure S1: Simulated cause-specific forces of mortality (true values)

2 Introducing stochasticity

We introduce artificial stochasticity in the simulated forces of mortality by generating random deviates from a Poisson distribution. To that aim, we take the average exposure for the US males between 1959 and 2010 from the same dataset that was used in the paper (Figure S2).

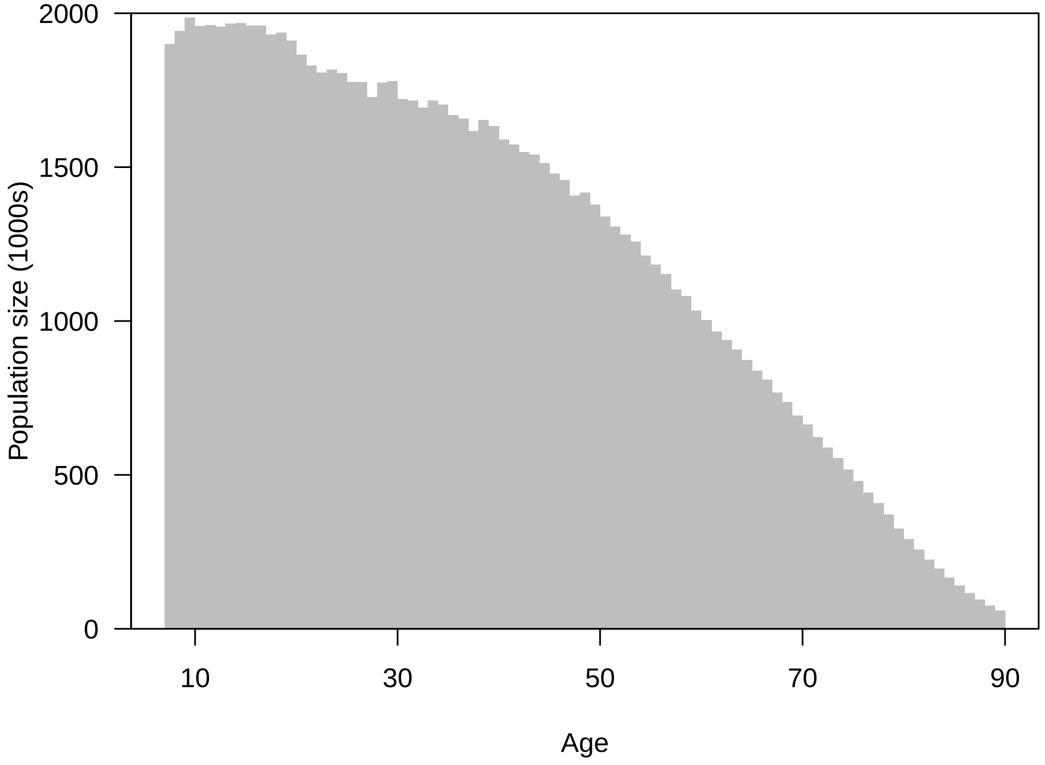

Figure S2: Population structure used as exposures to generate the stochastic noise

We simulate age- and cause-specific death counts (d_x^κ) from a Poisson distribution,

$$d_x^\kappa \sim \mathcal{P}(\lambda = n_x \, \mu_x^\kappa) \,, \tag{3}$$

which are then converted to age-specific rates (m_x^κ),

$$m_x^\kappa = \frac{d_x^\kappa}{n_x} \,, \tag{4}$$

where d_x^κ are the simulated death counts at age x from cause κ, n_x are the age-specific exposures, and μ_x^κ are the cause-specific forces of mortality. Figure S3 displays the simulated cause- and age-specific death rates.

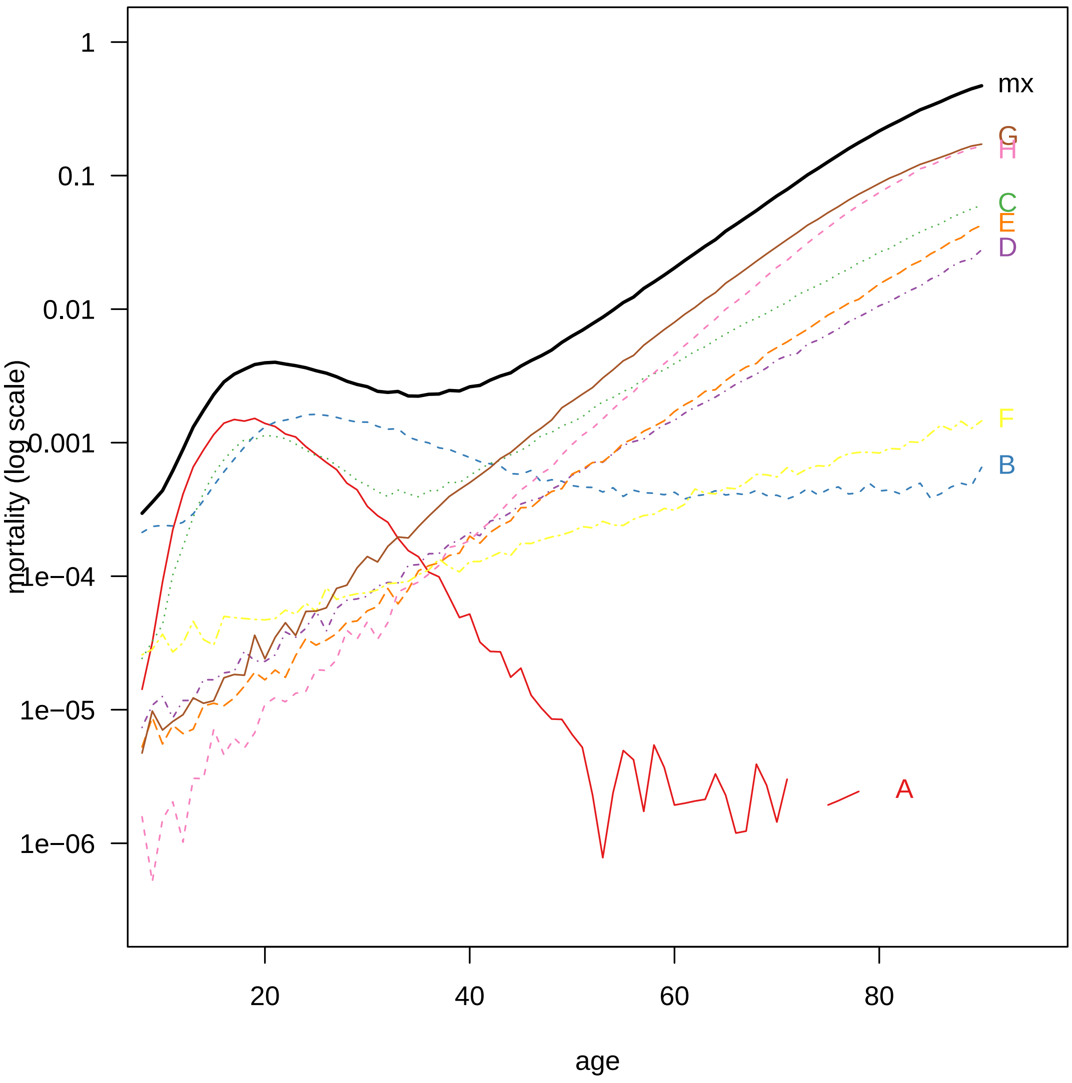

Figure S3: Simulated age- and cause-specific death rates

3 Brute force approach

The resulting age-specific death rates (m_x^κ) mimic real age- and cause-specific data gathered for a given population. It would be naïve to try to select the best candidate set based on all possible combinations of causes of death. In practice, this is often impossible since the number of combinations increases extremely fast with the number of causes in the dataset. For instance, working on the 92 causes included in the US data that are used in the paper, there would be $4.95 \cdot 10^{27}$ possible typologies, which correspond to all possible combinations of size 1 to 92. In this simulation with only 8 causes of death, there are only 255 such combinations, making a brute force approach feasible.

We thus run an unconstrained version of our model on all 255 possible sets of causes of size 1 to 8. Not constraining is essential at this stage because constraints could make even a poor choice of causes yield a reasonably good hump at the expense of the cause-specific fits[1]. We then compare the goodness-of-fit of each combination. We measure this fit by computing the residual sum of squares between the

[1]For instance, in the absence of true hump-contributing causes, other causes would be artificially attributed a con-

estimated age- and cause-specific contributions to the hump, and the true hump components of each cause. Algebraically, the residual sum of square (rss) is defined as

$$rss = \sum_{\kappa} \sum_{x} (\hat{\gamma}^{\kappa}_{H,x} - \gamma^{\kappa}_{H,x})^2 \,, \tag{5}$$

where $\hat{\gamma}^{\kappa}_{H,x}$ are the age- and cause-specific estimated contributions to the hump measured on the rate scale (i.e. not translated in units of life expectancy lost to the hump), and $\gamma^{\kappa}_{H,x}$ are the true cause-specific humps as defined in (2).

Figure S4 illustrates the fitted ($\hat{\gamma}^{\kappa}_{H,x}$) and true ($\gamma^{\kappa}_{H,x}$) contributions to the hump by cause of death for a selection of the 255 possible combinations. The top-left panel, ABC, corresponds to the best possible set, consisting in only causes that indeed have a hump component. This set shows very close correspondence between true and estimated contributions to the hump. This is the case even without constraining the sum of all cause-specific contributions to be equal to the all-cause hump, although there are some small residual negative contributions that would disappear with a constrained model[2].

tribution to the hump in order to make up for the overall hump. In the absence of empirical evidence for such a hump, this would make the identification of each component totally arbitrary and unstable. In practice, on this simulated data, our constrained model often does not converge when the choice of hump-related causes is too far from the true one. For this reason we cannot present a comparison of constrained and unconstrained results.

[2]Note that cause B is the one that displays the largest discrepancy between the true and the fitted contribution to the hump. This is due to the fact that of the three hump-contributing causes, this is the one for which the two mortality components (hump and senescence) are the furthest from their ideal-type (i.e. the hump is wide and the senescence component is almost flat, see Figure S1). This tends to make these components less identifiable and thus more difficult to split.

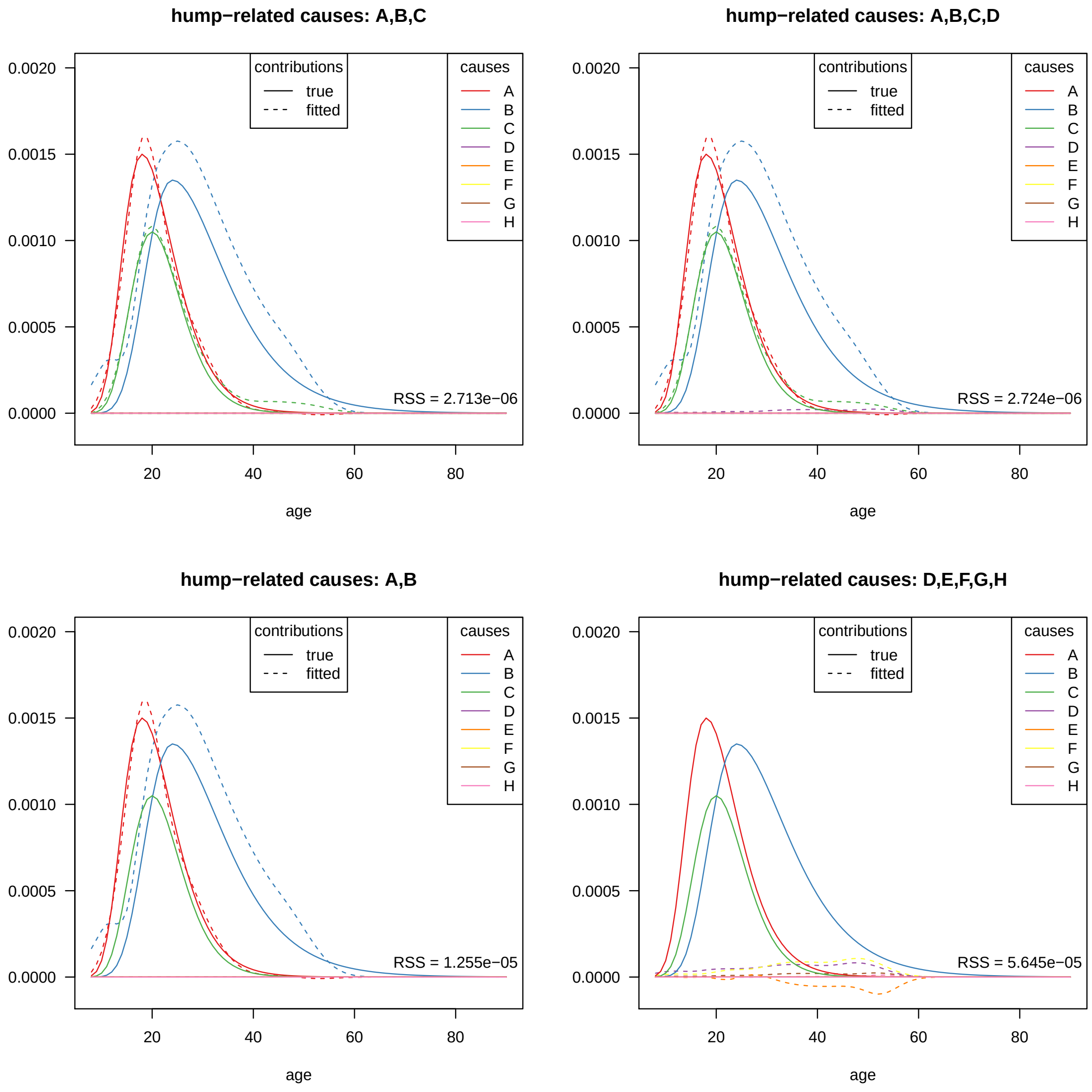

Figure S4: Fitted and true contributions to the hump for a selection of all 255 combinations

Table S2 shows that, out of the 255 possible sets of hump-contributing causes, the one that yields the best goodness-of-fit is the true case (i.e. that includes causes A, B and C only). The runner-ups are all based on augmentations of this set, with one or two additional causes. These "false positives", i.e. causes that were unnecessarily included in the list of contributors to the hump, do not imply much penalty on the quality of the fit. For example, the residual sum of squares of the 10th best combination (last one of Table S2) is only 6% higher than the first one.

	A	B	C	D	E	F	G	H	RSS
1	1	1	1						2.713E-06
2	1	1	1		1				2.714E-06
3	1	1	1	1					2.724E-06
4	1	1	1	1	1				2.726E-06
5	1	1	1				1		2.815E-06
6	1	1	1		1		1		2.816E-06
7	1	1	1	1			1		2.826E-06
8	1	1	1	1	1		1		2.828E-06
9	1	1	1			1			2.875E-06
10	1	1	1		1	1			2.876E-06

Table S2: Best combinations of causes in terms of goodness-of-fit. The causes that are included in the model are flagged with a "1" for each combination.

On the contrary, "false negatives", i.e. causes that are true hump contributors but were omitted from the decomposed set, have a much higher cost on the goodness-of-fit. Figure S5 indicates how the composition of the set of contributing causes changes from the best (left) to the worst-fitting (right) cases. The strong steps in this graph indicate that most of the quality of fit comes from the presence or absence of the causes that truly contribute to the hump (A, B and C). All 32 best solutions include causes A, B, and C. The next three steps are all of the sets that omit one of the three true contributors to the hump, first B omitted, then A, then C. The next three steps include all sets of causes that only include one of the true contributors (B, A, then C). Finally, the 31 worst solutions each omit all three true contributors. Within each of these steps, the cost of including a cause that in fact does not contribute to the hump is negligible. We conclude that it is preferable to include too many causes in the list of causes that might contribute to the hump than to inadvertently omit one. From the false positive heuristic, we conclude that our choice to use the same typology for both sexes in our analysis likely did not impose too heavy a penalty, despite the fact that women tend to count fewer causes that display a potential contribution to the hump.

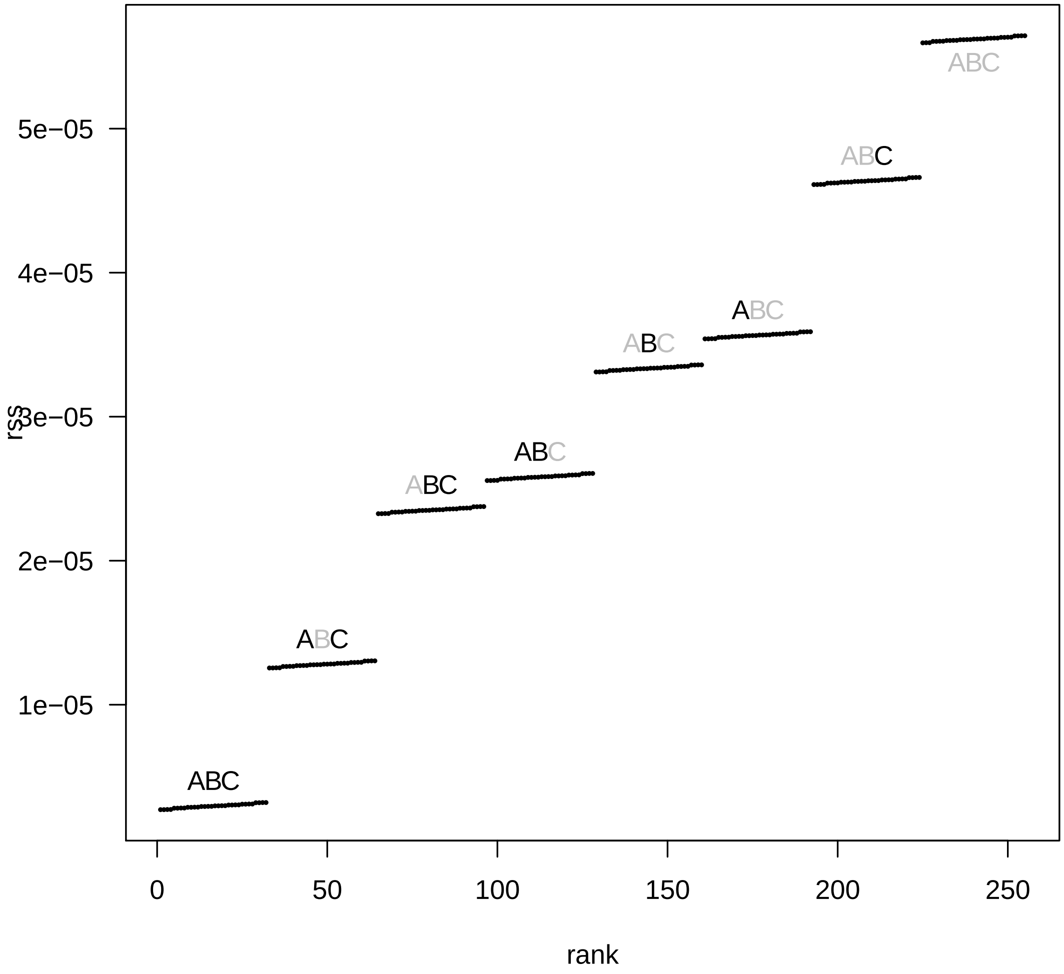

Figure S5: Ranking of all 255 sets of causes by their residual sum of squares. The presence or absence of a given cause A, B and C is indicated above each cluster of solution. Grey means a cause is absent, while black means it is present.

4 Inductive approach

Since in practice this exhaustive exercise is unfeasible, one needs an alternative procedure to identify the best set of candidate causes that contribute to the hump. We argue in the manuscript that inductive approaches, like Principal Component Analysis (PCA) or Cluster Analysis (CA), are efficient ways to achieve this task. In order to do that, we characterize each cause by its shape during the years that are mostly affected by the young adult mortality hump. A simple measure of this shape is the first difference of the observed death rates m_x^κ (which in our case are simulated):

$$\rho_{x+0.5}^\kappa = m_{x+1}^\kappa - m_x^\kappa \,. \tag{6}$$

The choice of the age range on which to apply this measure is arbitrary but should include the ages most affected by the hump. We will show however in the next section that this choice does not influence much the results. We first start here with the observations for ages below 40 years.

First we run a hierarchical clustering analysis on the euclidean distances between each ρ_x^κ in order to identify clusters of causes of death. Figure S6 shows that causes A, B and C clearly stand apart from the rest. Using the Average Silhouette Width (ASW) criterion (Kaufman & Rousseeuw, 1990), we determine that the ideal number of clusters is 4, with causes A, B and C constituting single-cause clusters, and a fourth composed of all other causes that do not contribute to the hump.

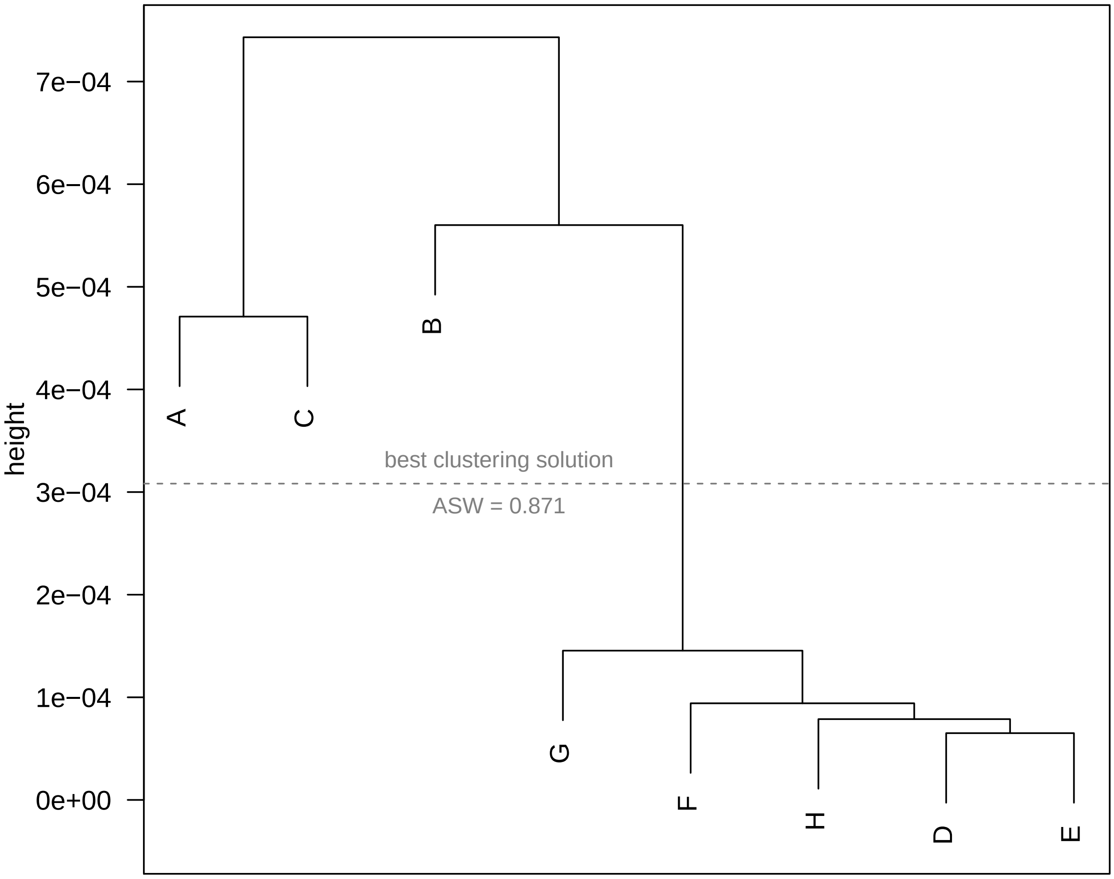

Figure S6: Results of Cluster analysis on the rate of ageing ρ^κ identify four clusters of causes of death that display distinctive shapes during early adulthood

We then run a PCA in order to decrease the dimensionality of the distance between each cause from 32 ages (8 to 39) to only two main dimensions. Figure S7 shows that these first two dimensions together explain 90% of the information. Once again, causes A, B and C visually stand apart. Black circles are drawn around each of the four identified clusters.

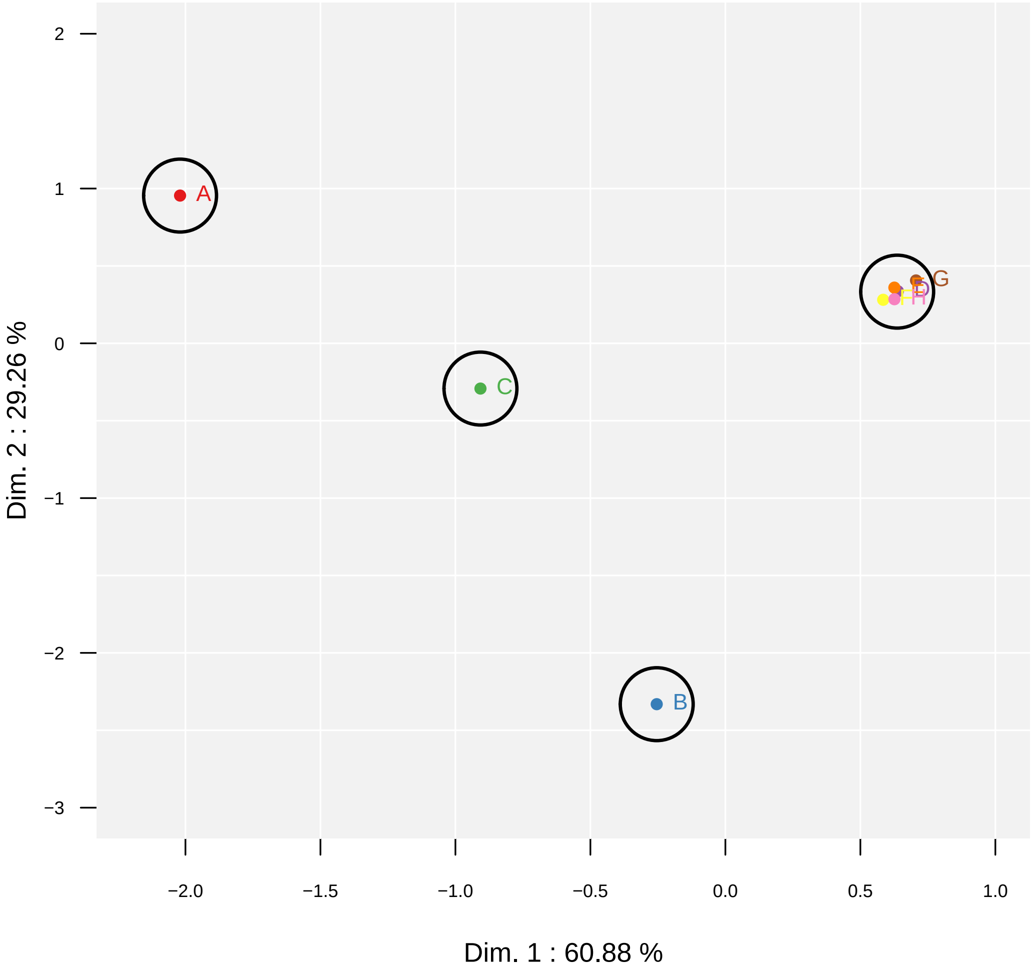

Figure S7: Results of Principal Component Analysis (right) shows that the four clusters clearly stand apart when reduced to their two principal dimensions

Together, these inductive analyses indicate that causes A, B and C are natural candidates for the cause-of-death decomposition of the young adult mortality hump. Since this set of causes corresponds to the true solution, this suggests that inductive approaches are a good shortcut to avoid the likely unfeasible task of testing all possible sets of contributing causes to the hump.

5 Choice of age boundaries

As stated previously, the choice of age bounds used for computing euclidean distances is partly arbitrary. The selection of causes by inductive approaches is however weakly affected by this choice. We demonstrate this by changing the age range on which we compute the first difference of the death rates, dropping progressively the top bound from age 39 to age 19. We then project these points on the original PCA. Figure S8 indicates that the relative position of each cause does not vary much with the choice of age range and that the resulting selection of causes that contribute to the hump would likely not be affected even if only ages 8 to 19 were included in the PCA

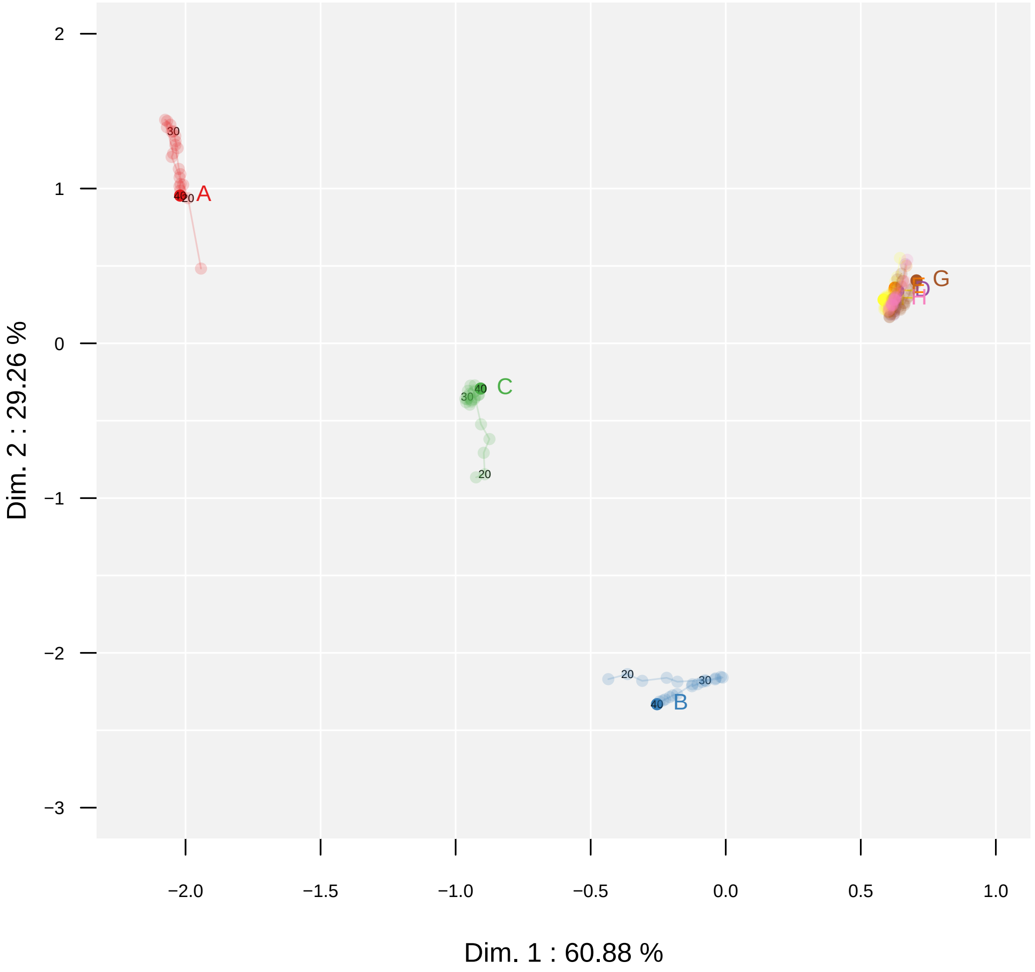

Figure S8: Influence of the choice of age range on the differences in shape of causes of death, as measured by the two first dimensions of a PCA run on the first difference in their age-specific death rates.

Conclusion

These sensitivity analyses support the robustness of our model to the choice of causes of death that contribute to the hump. First, we show that it is only weakly sensitive to false positives, i.e. the undue inclusion of causes that actually do no contribute to the hump. It is however more sensitive to false negatives, i.e. the omission of causes that actually do contribute to the hump. Cause-selection should therefore err towards overinclusion of causes. Second, we show that inductive approaches are an efficient way to identify the causes that contribute to the hump in the very common case where it is impossible to test all possible sets of causes. Third, we show that the choice of ages on which to apply these inductive approaches does not dramatically affect the choice of which causes to include in the decomposition.

References

Kaufman, L., & Rousseeuw, P. J. (1990). *Finding groups in data: an introduction to cluster analysis* (Vol. 344). John Wiley & Sons.

Kostaki, A. (1992). A nine-parameter version of the heligman-pollard formula. *Mathematical Population Studies*, *3*(4), 277-288.
